# Genetic mapping of *psl* locus and quantitative trait loci for angular leaf spot resistance in cucumber (*Cucumis sativus* L.)

**DOI:** 10.1007/s11032-018-0866-2

**Published:** 2018-08-17

**Authors:** Renata Słomnicka, Helena Olczak-Woltman, Aleksandra Korzeniewska, Dariusz Gozdowski, Katarzyna Niemirowicz-Szczytt, Grzegorz Bartoszewski

**Affiliations:** 1 Department of Plant Genetics Breeding and Biotechnology, Faculty of Horticulture Biotechnology and Landscape Architecture, Warsaw University of Life Sciences–SGGW, Warsaw, Poland; 2 Department of Experimental Design and Bioinformatics, Faculty of Agriculture and Biology, Warsaw University of Life Sciences–SGGW, Warsaw, Poland

**Keywords:** Cucumber angular leaf spot, Cucumber resistance, *Pseudomonas syringae* pv. *lachrymans*, *Psl*, QTL mapping

## Abstract

One of the most important cucumber diseases is bacterial angular leaf spot (ALS), whose increased occurrence in open-field production has been observed over the last years. To map ALS resistance genes, a recombinant inbred line (RIL) mapping population was developed from a narrow cross of cucumber line Gy14 carrying *psl* resistance gene and susceptible B10 line. Parental lines and RILs were tested under growth chamber conditions as well as in the field for angular leaf spot symptoms. Based on simple sequence repeat and DArTseq, genotyping a genetic map was constructed, which contained 717 loci in seven linkage groups, spanning 599.7 cM with 0.84 cM on average between markers. Monogenic inheritance of the lack of chlorotic halo around the lesions, which is typical for ALS resistance and related with the presence of recessive *psl* resistance gene, was confirmed. The *psl* locus was mapped on cucumber chromosome 5. Two major quantitative trait loci (QTL) *psl5*.*1* and *psl5*.*2* related to disease severity were found and located next to each other on chromosome 5; moreover, *psl5*.*1* was co-located with *psl* locus. Identified QTL were validated in the field experiment. Constructed genetic map and markers linked to ALS resistance loci are novel resources that can contribute to cucumber breeding programs.

**Electronic supplementary material:**

The online version of this article (10.1007/s11032-018-0866-2) contains supplementary material, which is available to authorized users.

## Introduction

Cucumber (*Cucumis sativus* L.) angular leaf spot (ALS), caused by the bacterium *Pseudomonas syringae* pv. *lachrymans*, is a common disease limiting open-field cucumber production. The symptoms of ALS include vein-limited, water-soaked lesions on leaves with or without a chlorotic halo, which later become necrotic. Water-soaked lesions can occur also on fruits, causing them to become misshapen and resulting in market yield reduction (Olczak-Woltman et al. 2008). The first study on cucumber resistance to ALS was published in 1964 by Chand and Walker. The authors defined disease severity as the number and size of the lesions and reported that the resistance is polygenetically inherited. By self-pollination and selection of the most resistant individuals, it was possible to accumulate genes conferring reduced severity, thereby increasing the level of ALS resistance (Chand and Walker 1964; Olczak-Woltman et al. 2008). Dessert et al. (1982) distinguished two types of cucumber ALS symptoms, either the occurrence of chlorotic halo around lesions typical for susceptibility or the lack of chlorotic halo related to resistance to ALS. The lack of chlorotic halo and resistance was conditioned by a single recessive gene *psl* (synonym *pl*) and is present in cucumber line Gy14 (Gy14A), commonly used in pickling cucumber breeding programs (Call and Wehner 2010–2011; Dessert et al. 1982).

Cucumber is a common vegetable and a model plant for genetic research in *Cucurbitaceae* due to relatively small-size genome and only seven pairs of chromosomes (Staub and Chung 2005). Recent progress in cucumber genome sequencing contributed tremendously towards mapping of the genes important for breeders (Huang et al. 2009; Pawełkowicz et al. 2016; Wóycicki et al. 2011; Yang et al. 2012). Several cucumber genetic maps have been constructed, and molecular markers linked to resistance or susceptibility genes for major cucumber diseases, including powdery mildew, downy mildew, scab, or viral diseases have been identified (Berg et al. 2015; Fukino et al. 2013; He et al. 2013; Nie et al. 2015a, 2015b; Sakata et al. 2006; Schouten et al. 2014; Wang et al. 2016; Wang et al. 2017; Zhang et al. 2013).

So far, there have been few attempts to find and map molecular markers linked to ALS resistance. A RAPD marker (OP-AO07_420_), linked to the *psl* locus, was identified at the distance of 13 cM in F_2_ mapping population developed from the cross of the susceptible inbred line B (B10) as the female with resistant line H 603 derived from *C*. *sativus* var. *hardwickii* as male parent (Olczak-Woltman et al. 2009). Despite this, reliable markers closely linked to ALS resistance genes in cucumber are lacking.

The aim of this study was to map *psl* locus and quantitative trait loci (QTL) controlling ALS severity to identify molecular markers that could facilitate efficient marker-assisted selection in pickling cucumber breeding programs.

## Materials and methods

### Mapping population

A population of 92 F_5:6_ recombinant inbred lines (RILs) was developed from a cross between inbred lines Gy14 and B10, using single-seed-descent. Previous studies confirmed that line Gy14 is resistant and B10 is susceptible to ALS (Olczak-Woltman et al. 2008). Gy14 was developed in the USA as a gynoecious line with white-spined fruits and the recessive *psl* resistance gene (Call and Wehner 2010–2011; Dessert et al. 1982). B10 is a highly inbred (> S_18_) Eastern-European cucumber line developed from cultivar “Borszczagowski,” monoecious with black-spined fruits, and susceptible to ALS (Olczak-Woltman et al. 2008). In each generation, sex expression (segregating gene *F*) and spine color (segregating gene *B*) were visually checked, and segregations were scored to confirm the random structure of the population. Gy14 and Gy14 × B10 F_1_ seeds were kindly provided by Prof. M.J. Havey (USDA-ARS Vegetable Crops Research Unit, Madison, WI, USA).

### Angular leaf spot resistance tests

The virulent *Pseudomonas syringae* pv. *lachrymans* strain 814/98 was used for plant inoculation (Olczak-Woltman et al. 2007). The genome of this strain has been recently sequenced (Słomnicka et al. 2018; GeneBank Accession No. NBLF00000000). All tests were performed as described earlier by Olczak-Woltman et al. (2008). To prepare inoculum, bacteria were grown on King B agar plates at 28 °C for 24 h. Bacterial colonies were suspended in sterile distilled water and adjusted to OD_600_ = 0.05 that is equal to the concentration of 1 × 10^7^ CFU ml^−1^. Plants were grown in the growth chamber conditions at 25 °C during the day and 22 °C at night, with 16 h photoperiod. At the 2nd to 3rd leaf stage, plants were inoculated by spraying the abaxial side of each leaf with inoculum prepared as described above. Sterile water was used as a control. Inoculated plants were kept in the darkness for 24 h at 22 °C, in relative humidity of 100%, and then for 6 days in the growth chamber under 16 h of light and with 90% relative humidity. Seven days after inoculation, plants were evaluated using 0/1 scale, where 0 means plants possessing lesions without a chlorotic halo, and 1 means plants with lesion halos. Parental lines and RILs were also scored using a nine-degree rating scale (Jenkins and Wehner 1983; modified by Olczak-Woltman et al. 2008) to calculate disease severity index (DSI), where 9 indicated none or single pin-point lesions, and 1 is for complete damage of leaves. Initially, parental lines (22 plants for each line), F_1_ plants (35), and F_2_ plants (267) were tested for resistance. After evaluation, infected leaves were removed, and plants were transplanted to the greenhouse and self-pollinated. In this experiment, the inheritance mode of the presence or absence of chlorotic halo was verified using chi-square goodness to fit test (*p* value < 0.05) (Supplementary File 1). The chlorotic halo heritability (h_2_) was estimated according to Olczak-Woltman et al. (2009). In the next two experiments, F_5_ and F_6_ generations of 92 RILs, together with parental lines as controls, were tested. Each line was tested using in total 16 plants (four replications with four plants in each). The DSI means obtained for each RIL in these two experiments were compared, and lack of statistically significant differences of DSI means was tested using Mann-Whitney *U* test.

### SSR and DArTseq genotyping

Total genomic DNA was extracted from leaves of young plants (stage of 3–6 leaves) both RILs and parental lines grown in plastic greenhouses using a GeneElute™ Plant Genomics MiniPrep Kit (Sigma-Aldrich, USA) according to manufacturer’s instructions.

The set of simple sequence repeat (SSR) markers for genotyping was derived from previously developed markers described by Ren et al. (2009), Yang et al. (2012), and Shen et al. (2015). All the markers were tested in silico for polymorphism by comparing of corresponding microsatellite regions in Gy14 and B10 genomic sequences. The set of 218 SSR markers, i.e., 86 SSRs of Ren et al. (2009), 113 of Yang et al. (2012), and 18 of Shen et al. (2015), were subsequently analyzed on parental lines and RILs (Słomnicka et al. 2015). Finally, the set of 52 high quality SSR markers was used for genotyping (Supplementary File 2). PCR amplification was performed using a PTC-200 thermocycler (Bio-Rad, USA) according to PCR program of Pillen et al. (2000). PCR was done in 20 μl reaction volume with DreamTaq Buffer, dNTPs Mix 2 mM, primers per 1 μM, 0.6 U of DreamTaq DNA Polymerase (Thermo Scientific, USA), and 40 ng of DNA. Amplicons were detected by electrophoresis on denaturating 6% polyacrylamide gel. The gels were silver-stained according the protocol of Benbouza et al. (2006), and amplicon segregations were scored.

The parental lines and 92 RILs in generation F_5_ were used for commercial DArTseq genotyping by sequencing that combined DArT technology (Wenzl et al. 2004) and Illumina HiSeq2000 sequencing (Illumina, CA, USA). In DArTseq method, genome-complexity reduction step is applied directing the analysis to hypomethylated, gene-rich genome regions. DNA is cut with two restriction enzymes, at least one of them is methylation sensitive, and genomic representations are obtained by ligation of adapters to the restriction fragments. The genomic representations are PCR-amplified and sequenced with the Illumina short read technology. DArTseq analytical pipeline is used to process the sequence reads and identify polymorphisms. DArTseq genotyping was performed at Diversity Arrays Technology Pty Ltd. (Canberra, Australia). DNA samples for DArTseq genotyping consisted 100 μl of 50 ng μl^−1^ DNA for each line. Obtained DArTseq data included two types of markers: SNPs and dominant in silico DArTs (Supplementary File 2).

### Linkage map construction

The SSR markers, SNPs, in silico DArTs, and morphological markers segregating in expected 1:1 ratio were used for genetic mapping. The markers segregation rate was verified using chi-square test (*p* value < 0.05). The linkage map of SSRs, SNPs, in silico DArTs, and morphological markers was constructed in Joinmap 4.0 (Van Ooijen 2006) (Supplementary File 3). To assign markers to linkage groups (LGs), Kosambi mapping function (Kosambi 1944) and logarithm of odds (LOD) of 8.0 as the minimum LOD score were used. LGs were identified based on markers anchoring on Gy14 genome and SSRs positions on previously constructed high-resolution cucumber genetic maps (Ren et al. 2009; Yang et al. 2012). MapChart 2.3 was used to prepare linkage group figures (Voorrips 2002).

### QTL identification

QTL analysis was conducted with MapQTL 5.0 software (Van Ooijen 2004). Preliminary, an interval mapping (IM) analysis to detect QTL, was performed. The possibility of QTL existence was scanned on every chromosome at intervals of 1 cM. Genome-wide LOD thresholds (*p* value < 0.05) were empirically determined for the trait using the permutation test (PT) of MapQTL with 1000 iterations. Based on the permutation tests, a threshold LOD value of 4.1 (α = 0.01) was used to declare the presence of a QTL. The multiple-QTL model (MQM) with selected markers as cofactors were performed to verify detected QTL. Each locus was named by an abbreviation of the trait followed by the chromosome number and locus number.

### Bioinformatics analysis

Using the reference cucumber genome of line 9930 (Huang et al. 2009), genomic regions corresponding to resistance loci that included 1 Mb flanking genomic regions were identified. The genes present in these regions were analyzed using Blast2GO and InterProScan package (Conesa et al. 2005) and cucumber genome 9930 v.2 annotation available at Cucurbit Genomics Database (http://cucurbitgenomics.org/organism/2).

### Validation of QTL in the field test

To validate the results, the field resistance test was performed. The experiment was performed at Wolica Experimental Station of the Department of Plant Genetics, Breeding, and Biotechnology (DPGBB), Warsaw University of Life Sciences–SGGW, Poland. Three-weeks-old seedlings of 92 F_6_ RILs were planted in field plots and artificially inoculated in the stage of three fully developed leaves. Eleven plants were tested for each RIL. After 1 week, DSI scoring was performed as described above. The markers that flanked both identified QTL loci, i.e., *psl5*.*1* and *psl 5*.*2*, were used to group RILs based on alleles—either from resistant line Gy14 or susceptible line B10.

## Results

### Evaluation of ALS resistance

**Fig. 1:**
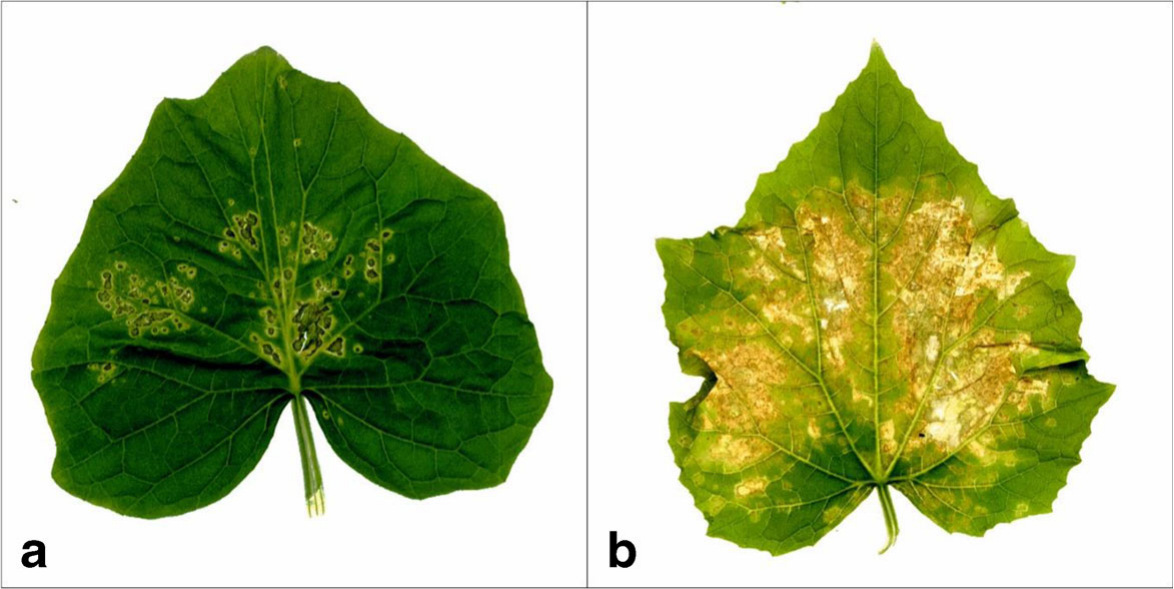
Angular leaf spot symptoms on leaves of cucumber lines Gy14 (**a**) and B10 (**b**) as result of inoculation with *P*. *syringae* pv. *lachrymans* strain 814/98 under growth chamber conditions, 7 days after inoculation

The paternal line B10 was susceptible to angular leaf spot. Initially widespread, water-soaked, angular chlorosis appeared on infected leaves that later became necrotic. Bacterial exudates on petioles and leaves were observed. Necrosis surrounded by an extensive chlorotic halo was always present. The DSI mean of B10 line was estimated to be 4.1 and 4.0 in two independent growth chamber tests, and the severity of symptoms was rated from over 50 to 75% of the leaf surface. The maternal Gy14 line possessed only small necrotic lesion on the leaves rarely surrounded by limited chlorosis. The disease symptoms observed for this line were similar to a hypersensitive response. The DSI mean of Gy14 plants varied between 6.3 and 6.5 in two independent growth chamber tests, and the symptoms appeared on 8 up to 25% of the leaf surface (Fig. 1, Table 1).

**Table 1:** The DSI for the parental lines and RILs of Gy14 × B10 population after inoculation with *Pseudomonas syringae* pv. *lachrymans* 814/98 under growth chamber (test 1 and test 2) and field conditions. *DSI*, disease severity index; *SD*, standard deviation

Test	Lines	Number of lines	DSI mean ± SD
Growth chamber test 1	Gy14	P_1_	6.3 ± 0.47
	B10	P_2_	4.1 ± 0.63
	F_5_ RILs Gy14-type	39	6.1 ± 0.27
	F_5_ RILs B10-type	47	4.2 ± 0.49
Growth chamber test 2	Gy14	P_1_	6.5 ± 0.49
	B10	P_2_	4.0 ± 0.62
	F_6_ RILs Gy14-type	41	6.1 ± 0.20
	F_6_ RILs B10-type	49	4.2 ± 0.41
Field test	Gy14	P_1_	7.3 ± 0.63
	B10	P_2_	4.6 ± 0.81
	F_6_ RILs Gy14-type	45	6.7 ± 0.30
	F_6_ RILs B10-type	39	4.8 ± 0.54

The plants of F_1_ generation demonstrated uniformly the same disease symptoms as B10 line, that is lesions surrounded by chlorotic halo were always present. In the F_2_ population, there were 190 plants with a chlorotic halo around lesions (*Psl*/−) and 77 plants without halo (*psl*/*psl*). Goodness of fit test confirmed the 3:1 segregation (${\chi}_{\mathrm{emp}}^2=2.1<{\chi}_{0.05}^2=3.84$, *p* value < 0.05). Thus, the chlorotic halo presence around the angular lesions is monogenetically inherited (Supplementary File 1). The estimated phenotypic, environmental, and genotypic variance was 1.2, 0.39, and 0.81, respectively. The heritability coefficient (h_2_) of angular leaf spot resistance expressed as presence or absence of a chlorotic halo was estimated to be 61.4% in the F_2_ generation.

The F_5_ and F_6_ RILs of mapping population Gy14 × B10 were tested for the presence of chlorotic halo surrounding lesions, and the scoring was consistent across both tests. Among 92 RILs, the lack of chlorotic halo, typical for Gy14 line, was observed for 39 RILs in F_5_ and for 41 RILs in F_6_; the intense chlorotic halo, typical for B10 line, was observed for 47 RILs in F_5_ and 49 RILs in F_6_. This segregation ratio equal to 1:1 was verified using chi-square test (Supplementary File 1).

In contrary to the monogenic inheritance of the chlorotic halo around lesions, the disease severity is polygenic. The DSI evaluation of RILs classified as Gy14-type ranged from 5.4 to 6.6 and from 5.6 to 6.5 in F_5_ and F_6_ populations, respectively. In case of B10-type RILs, DSI ranged from 3.3 to 5.3 and from 3.5 to 5.2 in F_5_ and F_6_ generations, respectively (Table 1, Supplementary Fig. 1). There were six RILs in the F_5_ generation and two RILs in the F_6_ difficult to score and thus, were not considered. The comparison of DSI mean performed by the Mann-Whitney *U* test between F_5_ and F_6_ populations for each RIL showed high convergence and no statistically significant differences between them.

### Molecular mapping and linkage map construction

In total, 714 molecular markers, including 52 SSRs, 330 SNPs, and 332 in silico DArTs, as well as 2 morphological markers (segregating *F* and *B* genes) and *psl* locus were used for linkage analysis and map construction. The map consisted of seven linkage groups corresponding to the seven cucumber chromosomes, included 717 loci, and spanned 599.7 cM. The average distance between markers was 0.84 cM. The linkage group length ranged from 46.5 cM for chromosome 7 to 111.1 cM for chromosome 4. The largest interval between two adjacent markers was 9.9 cM for markers IS_16325062 and 16326201 on chromosome 4. The number of markers for each chromosome was from 56 for chromosome 7 to 132 for chromosomes 3 and 4, respectively (Table 2, Supplementary File 3). Thus, obtained genetic map covered all chromosomes, and it was suitable for subsequent QTL mapping.

**Table 2:** A distribution of SSRs, SNPs, in silico DArTs, and morphological markers among seven cucumber chromosomes mapped with RIL population Gy14 × B10

Chromosomes	Map length (cM)	Number of loci	Average distance
Chr_1	88.7	97	0.91
Chr_2	89.3	88	1.01
Chr_3	78.7	132	0.60
Chr_4	111.1	132	0.84
Chr_5	78.6	123	0.64
Chr_6	106.8	89	1.20
Chr_7	46.5	56	0.83
Total	599.7	717	0.84

### *psl* locus mapping

**Fig. 2:**
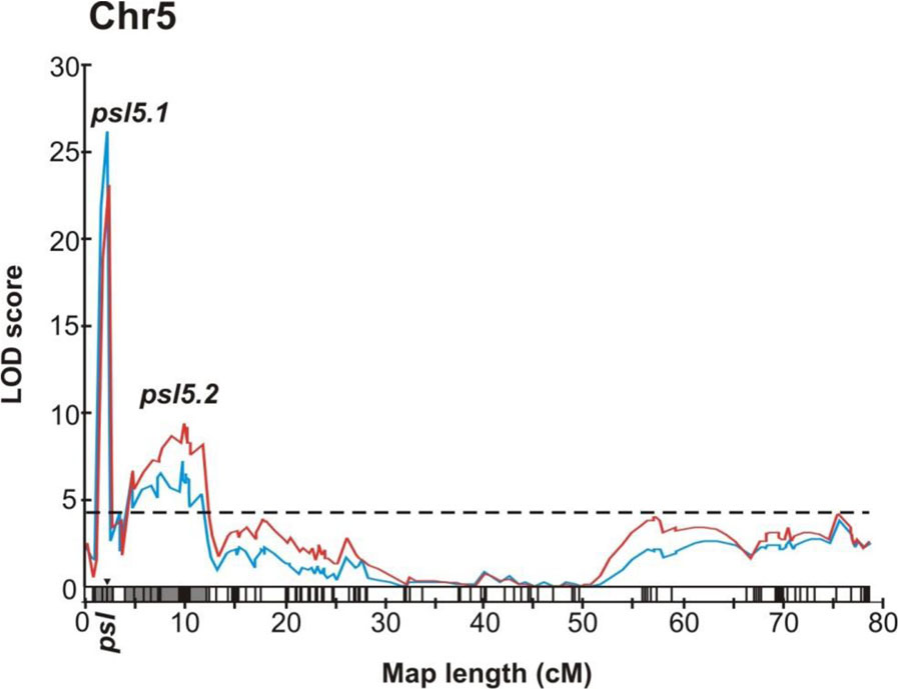
Positions of angular leaf spot resistance (ALS) loci at the linkage map derived from the F_5:6_ RILs population Gy14 × B10. Chromosome 5, which is carrying all identified ALS resistance loci, is shown. The *psl* locus is located at 2.0 cM. Two significant QTL *psl5*.*1* and *psl5*.*2* controlling ALS severity were detected. The *psl* locus is co-located with *psl5*.*1*. Red line is for growth chamber test 1, and blue line is for growth chamber test 2. LOD scores are shown on the y-axis, and the LOD threshold of 4.1 at *p* value < 0.05 on 1000 permutations is indicated by a horizontal dotted line

The phenotypic evaluation and genetic analysis confirmed monogenic recessive inheritance of *psl* locus as described by Dessert et al. (1982). Mapping of *psl* placed it on chromosome 5 at 2.0 cM. Two molecular markers, namely IS_16325300 and UW085415, located at the positions 1.6 and 2.4 cM on chromosome 5 were found to be the closest markers flanking *psl* locus with genetic distance of 0.4 cM (Fig. 2, Table 3, Supplementary File 3).

**Table 3:** Characteristics of the loci controlling angular leaf spot resistance. PVE% (*R*^*2*^), percentage of phenotypic variation explained by the QTL. The genomic positions of QTL intervals are based on cucumber line 9930 genome sequence

Locus	Genetic map interval (cM)	Flanking markers	LOD exp1/exp2	PVE% (*R*^*2*^) exp1/exp2	Additive effect exp1/exp2	Genomic location		
						Chromosome	Marker positions (bp)	Genomic region
*psl*/*psl5.1*	1.6–2.4	IS_16325300-UW085415	23.1/26.3	25.6/27.6	0.84/0.86	Chr 5	3,465,660-4,360,686	895 kb
*psl5.2*	4.7–10.5	16327616-IS_16326693	9.6/7.3	14.4/10.7	0.81/0.67	Chr 5	4,576,749-6,239,715	1.7 Mb

### Identification of QTL for ALS resistance

The DSI evaluation of ALS resistance from two growth chamber experiments was used for QTL mapping. Based on these experiments, two major QTL were identified on chromosome 5 and designated as *psl5*.*1* and *psl5*.*2*. Two other regions, at the positions 12.1–17.8 and 56.2–72.6 cM, with LOD score below the threshold were also detected (Fig. 2). The most prominent QTL with the highest LOD score was *psl5*.*1*. This QTL was flanked by two markers, i.e., namely IS_16325300 and UW085415, at the positions 1.6 and 2.4 cM, the same as for *psl* locus, thus they were co-located. The LOD score for this QTL was 23.1 and 26.3 explaining 25.6% and 27.6% of phenotypic variations for growth chamber tests 1 and 2, respectively. The second considerable QTL *psl5*.*2* was found also on chromosome 5 between SNP marker 16327616 and IS_16326693 at the positions 4.7 and 10.5 cM, respectively. The phenotypic variation which was explained by *psl5*.*2* was 14.4% and 10.7% with LOD score 9.6 and 7.3 for growth chamber test 1 and 2, respectively (Fig. 2, Table 3, Supplementary File 4).

The sequences of the markers flanking resistance loci were mapped to the cucumber reference genome 9930, and two genomic regions of chromosome 5 located next to each other were identified. The 0.8 cM genomic region flanked by the markers IS_16325300 and UW085415, carrying *psl*/*psl5*.*1*, was estimated to be 895 kb in size. The 5.8 cM genomic region of *psl5*.*2*, flanked by the markers 16327616 and IS_16326693, was 1.7 Mb in size (Table 3). In these genomic regions, 149 and 247 annotated genes were identified, respectively (Supplementary File 5).

### Validation of QTL

The field resistance test was performed to confirm the persistence of QTL *psl5*.*1* and *psl5*.*2*. Based on the markers flanking *psl5*.*1* and *psl5*.*2*, groups of RILs possessing alleles either from Gy14 or B10 line were identified. Thirty-eight RILs possessed Gy14 alleles for both flanking markers, while 44 and 45 lines possessed B10 alleles for IS_16325300 and UW085415 markers, respectively. The comparison of genotyping with DSI scoring showed that for IS_16325300 marker, 30 RILs from the Gy14 genotype group and for UW085415 marker, 32 RILs were classified as ALS resistant (about 80%), with field DSI mean of 6.9 and 6.7, respectively. In the group of RILs with B10 alleles for both markers, 31 RILs (about 70%) were susceptible with DSI mean of 4.9. For markers flanking *psl5*.*2*, namely 16327616 and IS_16326693, 44 and 43 RILs possessed Gy14 alleles, as well as 40 and 43 RILs possessed B10 alleles. The comparison of genotyping with DSI scoring showed that in Gy14 group, 36 RILs for marker 16327616 and 38 lines for marker IS_16326693 were ALS resistant (above 80%) with DSI mean of 6.8. In the group of RILs with B10 alleles, 31 and 33 lines (above 76%) were susceptible with DSI mean of 4.8 (Supplementary File 6).

## Discussion

Cucumber angular leaf spot is one of the main biotic stresses in field production of this worldwide common vegetable. Therefore, discovery and cloning of genes that confer resistance to ALS are critical to develop breeding strategies for better control of this disease. Here, we present the first cucumber genetic map with loci contributing to ALS resistance, both localized on chromosome five, at intervals 1.6–2.4 cM and 4.7–10.5 cM (Table 3). Several resistance genes and QTL for pathogens were localized on cucumber chromosome 5. In numerous studies, major QTL for downy mildew resistance (*dm*) were placed on chromosome 5, with *dm5*.*1* in very similar position to ALS resistance loci. In the study of Pang et al. (2013), *dm5*.*1* position was 0–25.7 cM, overlapping both ALS resistance loci. In other studies, *dm5*.*1* as well as *dm5*.*2* and *dm5*.*3* were located downstream to ALS resistance loci (Szczechura et al. 2015; Wang et al. 2016; Wang et al. 2017; Win et al. 2017; Yoshioka et al. 2014; Zhang et al. 2013). Also, QTL for powdery mildew and gummy stem blight resistance were found on chromosome 5, however at the other end of chromosome 5 (Berg et al. 2015; He et al. 2013; Liu et al. 2017; Nie et al. 2015a, 2015b; Xu et al. 2016). All those findings support the hypothesis of “resistance gene cassettes” located on chromosome 5, based on cucumber breeders observation that the resistances to major cucumber diseases are clustered and inherited together.

In this study, Gy14 × B10 RILs population was developed and tested to ALS resistance. We confirmed that the type of lesion is determined by the single recessive *psl* locus, and we mapped this gene on cucumber chromosome 5. Quantitative analyses resulted in major QTL *psl5*.*1* and *psl5*.*2* identification. Interestingly, *psl5*.*1* was co-located with *psl* locus, and *psl5*.*2* was located next to it. Chand and Walker (1964) indicated that ALS resistance estimated by lesion number and size may be controlled by two or more genes. As described by Dessert et al. (1982), Haley and Palmer (1977) found high positive correlation between ALS resistance and the type of lesions; lesions not surrounded by chlorotic halos were important component of the resistance. This was further investigated by Dessert et al. (1982), and a single recessive gene *psl* (*pl*) was found to control lesions type. Later, Olczak-Woltman et al. (2009) showed that non-halo lesion-type reaction to *P*. *syringae* pv. *lachrymans* infection, that is similar to hypersensitive response, contributes to ALS resistance. Here, we confirmed these studies, showing that the lack of halo surrounding lesions is conferred by the single recessive *psl* locus that is co-located with *psl5*.*1*, and it is an important compound of ALS resistance.

The constructed genetic map possesses seven linkage groups which are in accordance with seven cucumber chromosomes. Because only few polymorphic SSR markers were detected, DArTseq genotyping was required to identify satisfactory number of polymorphic markers. DArTseq is a type of genotyping by sequencing  method in which genome-complexity reduction step is applied in order to direct the analysis to the hypomethylated, gene-rich genome regions. DArTseq worked well in our study, delivering up SNPs and presence/absence of variation markers (in silico-DArTs). All the markers were identified for very narrow cross, making the markers more useful for practical breeding because polymorphisms should exist in elite germplasm. SSRs order was highly consistent with their location on the maps developed by Ren et al. (2009) and Yang et al. (2012). SNPs and in silico DArTs order was consistent with cucumber reference genome sequence. Chromosomal locations of *B* and *F* genes (morphological markers) were in agreement with previous studies (Li et al. 2013; Miao et al. 2011). The *psl* locus and *psl5*.*1* QTL were co-located and mapped at the 0.8 cM interval flanked by markers IS_16325300 and UW085415. The QTL *psl5*.*2* was flanked by markers 16327616 and IS_16326693 in 5.8 cM interval next to the first one. The genomic regions carrying ALS resistance loci spanned about 895 kb and 1.7 Mb in size, respectively. These relatively large size of genomic regions corresponding to QTL can be related to limited genetic diversity of this region. It was found that one of the cucumber domestication-related genomic regions, carrying bitterness locus *Bt*, is located on chromosome 5, and it was characterized by limited nucleotide diversity (Qi et al. 2013). Also, the mapping population used in this study was developed from relatively narrow cross of two-picking cucumber lines Gy14 and B10. Limited diversity within the ALS resistance loci can be related to Gy14 line development and introgression of genomic segment(s) from female accession that could negatively affect recombination frequency.

The identification of QTL stable across multiple environments and populations plays essential role in later marker-assisted selection (Ma et al. 2017). Two identified in this study QTL were verified in field conditions. The *psl5*.*1* and *psl 5*.*2* were validated by comparison of flanking markers (IS_16325300, UW085415, 16327616, and IS_16326693) genotyping with DSI results in the field test. Based on the genotyping, RILs of mapping population were classified as representing Gy14 and B10 alleles. The comparison showed compatibility of these results in over 80% for the group of RILs with Gy14 alleles and in over 70% for the group of RILs with B10 alleles, which is relatively high. Thus, the markers flanking *psl5*.*1* and *psl5*.*2* loci may be suitable to support selection for angular leaf spot resistance in pickling cucumber-breeding programs.

## Electronic supplementary material

Supplementary Figure S1 Frequency distribution of disease severity index to angular leaf spot in the Gy14 × B10 cucumber RIL population. (**a**) growth chamber test 1, (**b**) growth chamber test 2, (**c**) field test. (DOCX 149 kb)

Supplementary File S1 Genetic analysis of lesions surrounded by chlorotic halo on cucumber leaves inoculated a with *P. syringae* pv. *lachrymans* under growth chamber conditions*. (XLSX 10 kb)*

Supplementary File S2 Lists of SSRs, DArTSeq SNPs, and in silico DArT markers used in this study. (XLSX 70.6 kb)

Supplementary File S3 List of loci placed on the genetic map and marker positions. (XLSX 41 kb)

Supplementary File S4 Detailed description of QTL identified on chromosome 5. (XLSX 16 kb)

Supplementary File S5 Genomic region of the QTL intervals. (XLSX 40 kb)

Supplementary File S6 Comparison of *psl/psl5.1* and *psl5.2* flanking markers genotyping and DSI scoring. (XLSX 9 kb)
