# Supplementary material for: Genetic mapping of psl locus and quantitative trait loci for angular leaf spot resistance in cucumber (Cucumis sativus L.)
Source: Mol Breed. 2018 Aug 17;38(9):111. doi: 10.1007/s11032-018-0866-2 (PMC6105252; doi:10.1007/s11032-018-0866-2)
Supplement: Supplementary file 1 [file 11032_2018_866_MOESM1_ESM.docx]

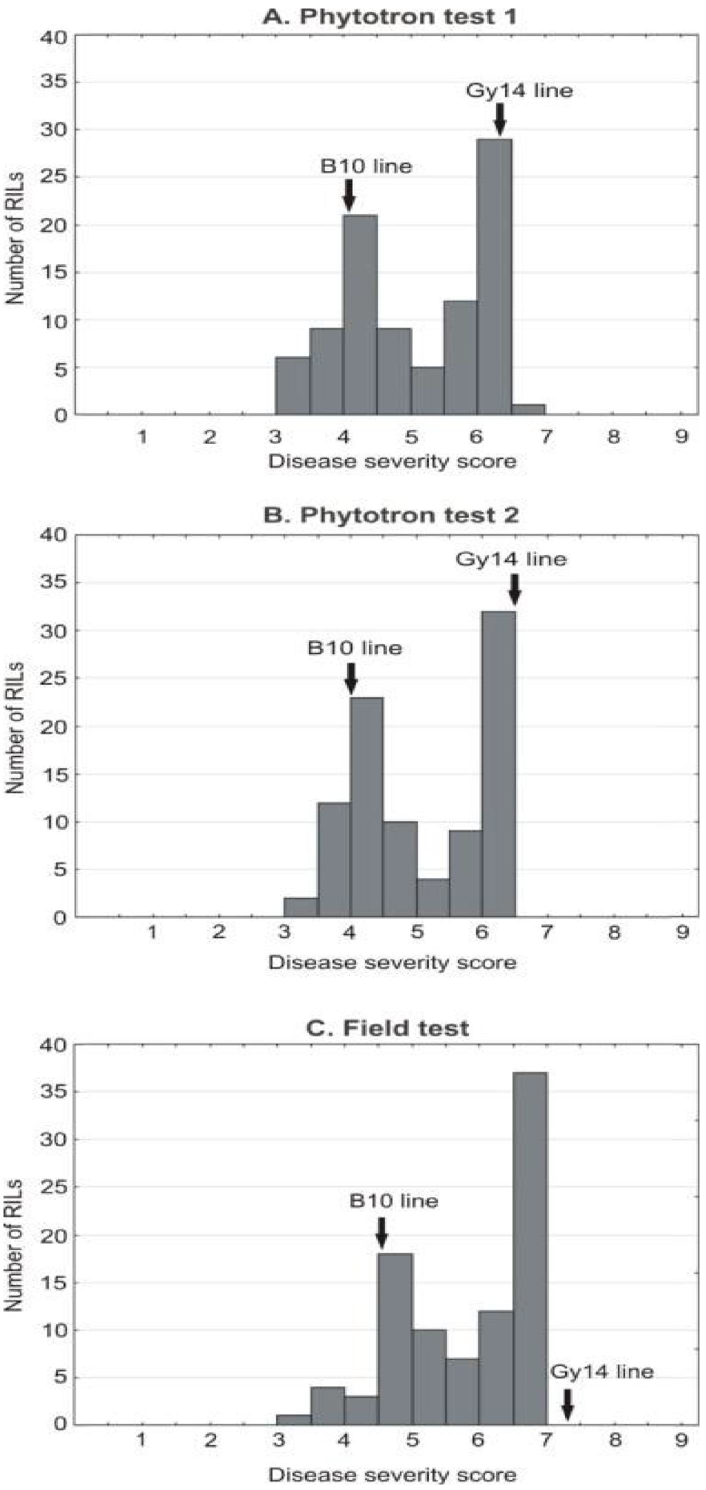
A. Phytotron test 1
Gy14 line
B10 line
Number of RILs
Disease severity score
B. Phytotron test 2
Gy14 line
B10 line
Number of RILs
Disease severity score
C. Field test
B10 line
Gy14 line
Number of RILs
Disease severity score
